# Mammography stages of change in middle-aged women with schizophrenia: An exploratory analysis

**DOI:** 10.1186/1471-244X-6-49

**Published:** 2006-10-30

**Authors:** Laurie A Lindamer, Emily Wear, Georgia Robins Sadler

**Affiliations:** 1 Department of Psychiatry, University of California, San Diego, 9500 Gilman Drive #0603, La Jolla, CA, 92093-0603, USA; 2 VISN 22 Mental Illness Research, Education and Clinical Center (MIRECC), VA San Diego Healthcare System, 3350 La Jolla Village Drive, San Diego, CA 92161, USA; 3 Department of Surgery, University of California, San Diego, 9500 Gilman Drive #0850, La Jolla, CA, 92093-0850, USA; 4 Rebecca and John Moores UCSD Cancer Center, 3855 Health Sciences Drive, La Jolla, CA 92093, USA

## Abstract

**Background:**

Health care providers and educators who seek to create health promotion programs and individualized comprehensive care plans for women with schizophrenia are hindered by the lack of data to guide their efforts.

**Purpose:**

This study tested the hypothesis that women with schizophrenia adhere to mammography screening guidelines at the same rate as other same-age women. The study also investigated the validity of the Health Belief (HB) and Stages of Change (SOC) models for breast cancer screening among women with schizophrenia.

**Methods:**

Socio-demographic and clinical variables, as well as knowledge, attitudes, and barriers were assessed as a function of stage of change related to breast cancer screening in 46 women with schizophrenia.

**Results:**

Women with schizophrenia were statistically less likely to be adherent to the screening recommendations than those without schizophrenia. Some support was found for the validity of the HB and SOC models for breast cancer screening in women with schizophrenia. Women in the Precontemplation stage had significantly higher negative attitude scores compared to Contemplation and Action/Maintenance stages (59.7, 45.7, and 43.2, respectively), and there was a trend for more barriers in the Precontemplation group (4.6, 2.6, 2.7 respectively).

**Conclusion:**

Given the small sample size, further research on the rates of breast cancer screening in women with schizophrenia is warranted. Nonetheless, these data suggest that providers who care for women with schizophrenia may need to make take additional measures to ensure that this population receives appropriate screening so as to not put them at greater risk for a late-stage diagnosis of breast cancer. Furthermore, these pilot data suggest that HB and SOC theory-based interventions may be valid for increasing mammography rates in women with schizophrenia.

## Background

In the last decade, the overall incidence of breast cancer has remained stable, while mortality has decreased [1]. Some, but not all, studies showed that rates of late-stage diagnoses have decreased [2-5]. At least a portion of the success in reducing breast cancer-related morbidity and mortality can be attributed to the early detection afforded by regular use of screening mammography among women 40 and older. Although breast cancer screening has been steadily increasing since the late 1980's and overall rates are approaching the defined targets [i.e., Healthy People 2010 [6]], use of mammography in some groups of women is still low [1]. Underserved women include those who are poor, less educated, non-Caucasian, living in rural areas [7], lacking health insurance or a usual source of care, physically challenged [8], coping with cognitive limitations [9], and diagnosed with severe and persistent psychiatric disorders, such as schizophrenia and related psychotic disorders [10,11].

Researchers have developed specific interventions to increase breast cancer screening in many of these under-represented groups [12]. However, there remains a relative lack of studies investigating the breast cancer screening rates of women with psychotic disorders. Little is known about the knowledge and benefits of, attitudes toward, and barriers to mammography in women with schizophrenia, and there is a similar paucity of interventions to promote screening in this group [13].

Moreover, older women with psychosis may be at increased risk for breast cancer because of factors related to their psychiatric disorder or its treatment. Some [14-16], but not all [17-21], studies have found an increased risk of breast cancer in women with schizophrenia, the reasons for which are not yet clear. This may, in part, be due to the fact that women with schizophrenia are more likely to have the general risk factors commonly associated with increased incidence of breast cancer, such as nulliparity, obesity, high fat diet, or physical inactivity [22,23]. In addition, there may be factors specifically related to schizophrenia and its treatment that increase risk for breast cancer [24]. Barriers to medical care, such as fear of condemnation, stigma, and limited finances, also restricted access to early detection and treatment among women with schizophrenia [25].

To understand the use of mammography, researchers have employed constructs from theories of health behavior change, such as the Health Belief (HB) model [26] and the Stages of Change (SOC) model [27]. The Health Belief model [28] considers health behavior a result of the interplay among variables that include perceived susceptibility to illness, perceived severity of illness, perceived benefits of taking health action, perceived barriers to taking action, and repetitive cues to health action. According to this model, women are more likely to undergo mammography if they believe that they are susceptible to breast cancer, consider its consequences severe, are aware of the benefits of screening, perceive that the benefits outweigh the barriers, and receive repeated cues to be screened. Perceived susceptibility and perceived barriers are usually the most important predictors of preventive health behavior, including mammography use [29,30], and most, but not all, studies testing the HB model in mammography have supported the model [30-42].

The Stages of Change Model has also been useful in understanding breast cancer screening. Specifically, the stages of adoption of the behavior and decisional balance have been used to predict rates of mammography in the general population [43-45]. The model proposes that people pass through a series of progressively more committed stages in the course of changing a health-related behavior: Precontemplation (not even thinking about the target behavior), Contemplation (currently not doing the behavior, but considering the adoption of the behavior), Action (beginning to adopt the behavior), and Maintenance (sustaining the behavior over time). An algorithm was developed for mammography stages of change that included these five stages, as well as two others, Relapse Precontemplation, and Relapse Contemplation that differ from the Precontemplation and Contemplation stages, in that women in these categories have had a mammogram in the past, are not currently on schedule, and may or may not be considering undergoing mammography in the next six months [46,45]. In this algorithm, the criteria for scoring positively (having a mammogram) were age dependent: women age 40–49 were expected to be screened at least every two years and women aged 50 and older, yearly. Several investigators have applied the concept of stages of change to assessing the efficacy of interventions to improve mammography adherence [44,47,48].

Decisional Balance, another construct of the SOC model, has been applied to mammography. It is a summary index derived from perceived positive (pros) and perceived negative (cons) features of the target behavior [49]. The model hypothesizes that people in the Action and Maintenance stages have a positive decisional balance (pros>cons) and that people in Pre-contemplation have a negative balance (cons>pros). Persons in Contemplation have a decisional balance that falls between Pre-contemplation and Action and are expected to be closer to neutral or zero point of equal pros and cons.

To date, there has been limited application of the SOC model to promoting recommended health behaviors in persons with schizophrenia. Some have examined stages of change to address alcohol use in schizophrenia with mixed results [50-52]. Others have reported validity of the model with respect to smoking cessation in schizophrenia [53]. Some have criticized the application of SOC model in persons with schizophrenia because some of these individuals lack several of the essential characteristics posited by the theory that are necessary to change behavior–namely, motivation and self-control, cognitive and behavioral coping skills, and social support [54]. The success of the SOC model, however, may be dependent on the behavior targeted for change and the characteristics of the sample. For example, eliminating addictive behaviors, such as substance use, may be very different from promoting preventive health behaviors, such as increasing the use of mammography. Furthermore, dually diagnosed persons (schizophrenia and substance use disorder) often have the more severe symptoms [55], poorer medication compliance [56], and increased use of institutional and emergency services [57,58] than non-substance addicted persons, suggesting that these individuals are not representative of all persons with schizophrenia.

At least one study has examined constructs of the HB and SOC models jointly for mammography use in a predominantly Caucasian middle class population. Champion and colleagues [36] found that women in the Action and Maintenance stages perceived significantly higher susceptibility, more seriousness, fewer barriers, and more benefits than those in the Pre-contemplation or Contemplation stages. No studies were found that described the relationship among knowledge and benefits of attitudes toward, and barriers to, breast cancer screening and stage of change in middle-aged women with schizophrenia, a group that possesses many risk factors for breast cancer.

The purpose of this study was to test the following three hypotheses: 1) women with schizophrenia are less likely to adhere to recommended screening guidelines than other same-age women; 2) the Health Belief and Stages of Change models will be a viable models for predicting breast cancer screening among women with schizophrenia; and 3) women with schizophrenia who are classified as being in the Precontemplation or Contemplation stage will have lower scores on their test of knowledge and benefits, more barriers to cancer screening, and more negative attitudes toward cancer screening (more negative decisional balance scores) than women with schizophrenia in the Action or Maintenance stages, a pattern that is typical in the general population.

## Methods

### Participant Eligibility

Women were eligible for the study if they had a DSM-IV [59] diagnosis of schizophrenia or schizoaffective disorder, were at least 40 years of age, were community-dwelling outpatients under the care of a psychiatrist, had no known diagnosis of dementia, had a history negative for breast cancer, were psychiatrically and medically stable, and were able to give informed consent.

### Informed Consent

All women gave written, informed consent, following a protocol approved by the researchers' Institutional Review Board. Trained research assistants met with each woman to conduct the interviews and to administer verbally the five survey instruments. All data were based on self-report unless the participant could not provide key information, such as date of last mammogram. In these cases, medical records were reviewed with the subject's consent.

### Survey Instruments

#### Socio-demographic and Clinical Information

Socio-demographic information, medical history, and gynecology service use were gathered from face-to-face interviews. The Positive and Negative Syndrome Scale (PANSS) [60] was used to assess positive and negative symptoms of schizophrenia, as well as general psychopathology.

#### Stage of Change for Mammography

The stage of change definitions for mammography followed the age-based recommendations; at least every two years for ages 40–49 and yearly for age 50 and older [44,46]. Precomtemplation stage consisted of women who never had a mammogram and were not considering receiving one; whereas Relapse Contemplation included women who had undergone mammography in the past but had not had one in the recommended time frame and were not considering having one. Women were classified as being in the Contemplation stage if they had never had a mammogram but were considering undergoing mammography, and similarly they were categorized as Relapse Contemplation if they had a mammogram in the past but not within the recommended time frame and were considering having one. Action stage was defined as being adherent with the screening recommendations, and Maintenance stage was defined as having had more than one mammogram according to the recommendations.

#### Knowledge toward and Benefits of Breast Cancer and Barriers to Screening

Knowledge and benefits about breast cancer and mammography were assessed using the questionnaire developed by Skinner and colleagues [61]. It consisted of eight true/false items about breast cancer knowledge and five items about perceived benefits of mammography. A total score was computed by summing the number of correct responses to both the knowledge and benefit items for a maximum score of 13.

The measures developed by Rakowski and colleagues [49] were used to measure decisional balance for mammography. It is a summary index that quantifies the net results of balancing of pros and cons for adopting a new health behavior. The women rated their agreement according to a 5-point Likert-type scale (1 = strongly disagree to 5 = strongly agree) with each of six "pro" statements and seven "con" statements for mammography. An index of decisional balance was calculated by transforming the "pro" index and "con" index for the mammography scales into percents, and subtracting the "con" percent from the "pro" percent. Positive decisional balance scores indicate a favorable assessment of pros versus cons (historically characteristic of a person already performing the behavior); negative values indicate a more unfavorable assessment (historically characteristic of persons not yet committed to the behavior). Decisional balance of about zero represents a mixed perspective of positive and negative opinions, which is typical among persons contemplating the behavior.

The Health Belief model purports that a person also considers the barriers in deciding whether or not to adopt a new health behavior. Some barriers are attitudinal, while others are instrumental (e.g., cost, transportation). The heterogeneity in the types of barriers necessitates that they be examined separately from knowledge (perceived susceptibility, severity and benefits) or attitudes (decisional balance). Participants also responded to a list of 15 items representing possible barriers to mammography that was used by Skinner and colleagues [46]. The total score was the total number of items positively endorsed.

### Analysis

Descriptive characteristics of the sample by stage of change were analyzed using Chi-square tests for categorical variables and analysis of variance (ANOVA) for continuous variables. Separate ANOVAs were conducted for each of the dependent variables (total score on the test of knowledge and benefits; total percent positive attitude, total percent negative, the decisional index, and number of barriers), and Spearman rho correlations were conducted to assess the relationship between the dependent variable and stage of change. Post-hoc analysis of individual items of the Decisional Balance and Barriers questionnaires for mammography were conducted using Chi-square tests. For this analysis, the 5-point Likkert scale of the Decisional Balance questionnaire was collapsed into two categories: agree and disagree. All tests were two-tailed with alpha set at 0.05. The slightly increased experiment-wide Type I error that might result by using an alpha of 0.05 for each dependent variable, rather than alpha of 0.01 (Bonferroni corrected), outweighed the possible Type II error, given the importance of understanding knowledge and benefits of attitudes toward, and barriers to, breast cancer screening and developing interventions to reduce risk for breast cancer for this group.

## Results

Subjects were a sample of convenience and consisted of 46 women between the ages of 44 and 72 years with a mean age of 52.9 (SD = 6.0 years). They were predominantly Caucasian (80%), and all were participating in on-going studies at a university-affiliated research center. The majority (65%) had never been married, and they averaged 12.3 (± 1.9) years of education. Most participants (73.3%) resided in an assisted care facility.

Nearly all of the women (88.8%) reported that they had insurance, a primary care physician (91.1%), and annual physical examinations (73.3%). Less than half of the women reported receiving one or more gender-specific preventive screenings within the past year: pelvic examination (45.7%), Pap test (43.5%), or mammogram (41.3%), and just over one third received none of the screenings. Twenty eight percent of the women were under the age of 50. Since the screening guidelines are less rigorous for this age group, the mammography rates for women under age 50 were examined separately. Table 1 shows that more of the younger group reported having mammograms than the older group; therefore, we combined both age groups for analysis. To compare annual rates of mammography in women with schizophrenia to reported rates in 40–64 year old women in California, the data were analyzed using the same aged subjects. Thirty seven percent of the women with schizophrenia reported having an annual mammogram.

**Table 1:** Means (SD) or percentages for socio-demographic, clinical, and service use by stage of adoption in older women with schizophrenia.

	**Precontemplation (n = 10)**	**Contemplation (n= 7)**	**Action/Maintenance (n = 29)**	**F or χ^2 ^Value**	**p-value**	
**Age (years)**	52.7 (4.4)	52.3 (6.8)	53.1 (6.4)	2.34	.940	--
**Ethnicity (% Cauc.)**	90.0	85.7	75.9	1.65	.800	--
**Marital Status (% ever married)**	60.0	28.6	75.9	5.71	.057	--
**Education (years)**	13.1 (2.5)	11.3 (0.95)	12.2 (1.8)	2.00	0.149	--
**Living Situation (% Assisted Living)**	80.0	71.4	69.0	1.12	.892	--
**PANSS Positive**	15.8 (5.5)	12.3 (5.6)	11.9 (4.6)	2.38	0.105	--
**PANSS Negative**	15.9 (4.7)	11.9 (2.9)	12.4 (4.7)	2.56	0.089	--
**PANSS General**	29.7 (7.42)	25.0 (3.4)	25.0 (5.8)	2.51	0.093	--
**Insurance (%yes)**	80	100	89.2	1.68	.432	--
**Primary Care Provider (%yes)**	70.0	100	96.6	7.15	.028	P<C, A/M
**Annual Physical Exam (%yes)**	30	42.9	96.4	21.35	.000	P, C<A/M
**Annual Pelvic Exam (%yes)**	20	28.6	58.6	5.44	.066	--
**Annual Pap (%yes)**	20	28.6	55.2	4.49	.106	--
**Annual Mammogram (%yes) Age≥50 years**	0	0	60.0	12.26	.002	P, C<A/M
**Annual Mammogram (%yes) Age<50 years**	0	0	77.8	6.74	.034	P, C<A/M

Precomtemplation; C = Contemplation; A/M = Action/Maintenance

Using the algorithm developed by Skinner and colleagues [46], only 4.3% (n = 2) of women with schizophrenia were classified as being in the Precontemplation stage; while, 17.4% (n = 8) were rated as being in the Relapse Precontemplation stage. None of the women met the definition for the Contemplation stage, 15.2% (n = 7) were categorized as being in Relapse Contemplation, 13.1% (n = 6) met criteria for the Action and 50% (n = 23) were classified as being in the Maintenance stage. Given the small sample sizes, the Precontemplation stage was combined with the Relapse Precontemplation stage (n = 10) and the Action stage was combined with the Maintenance stage (n = 29). Relapse Contemplation remained a separate category (n = 7). Three groups were created: 1) women who were not considering having a mammogram in the future whether or not they had one in the past (Precomtemplation, 21.7%); 2) women who underwent mammography more than two years ago, who were considering having another (Contemplation, 15.2%); and 3) women who had a mammogram in at least the past two years and who regularly underwent age-appropriate screening (Action/Maintenance, 63%).

Results of ANOVAs revealed that there were no significant differences among the groups on knowledge and benefits scores or number of barriers, although the barriers to receiving mammograms reported by the women differed at a trend level (p = 0.081; see Table 2). While the Positive Decisional Balance scores did not differ among the groups, Negative Decisional Balance scores demonstrated a significant difference among the stages. Women in the Precontemplation group had significantly more negative attitudes toward mammography than the other groups, although all groups demonstrated negative attitudes toward screening. The Decisional Balance Index also differed significantly between the Precontemplation and the other two groups. There were no significant differences among the stages of change on any of the demographic or clinical variables.

**Table 2:** Means (SD) on measures of breast cancer screening knowledge, barriers, and decisional balance by stage of adoption.

	**Precontemplation **(n = 10)	**Contemplation **(n= 7)	**Action/Maintenance **(n = 29)	**F-value**	**p-value**	**Group Comparisons**
**Knowledge and Benefits (total score)**	10.9 (1.6)	10.4 (2.2)	10.9 (2.7)	.101	.904	--
**Barriers (total score)**	4.6 (3.1)	2.6 (1.4)	2.7 (2.2)	2.670	.081	P>C, A/M
**Positive Decisional Balance (%)**	79.0 (20.1)	87.1 (15.1)	92.1 (25.8)	1.161	.323	--
**Negative Decisional Balance (%)**	59.7 (13.8)	45.7 (11.1)	43.2 (18.1)	3.794	.030	P>C, A/M
**Decisional Balance Index**	-19.3 (25.9)	-48.9 (29.1)	-41.4 (17.5)	4.452	.017	P<C, A/M

P = Precomtemplation; C = Contemplation; A/M = Action/Maintenance

To further examine the relationship between measures of decisional balance and stage of change, Spearman rho correlations were conducted. The Negative Decisional Balance score, and consequently the Decisional Balance Index, were found to be significantly inversely associated with stage of change (rho = -.394, p = .007 and rho = -.391, p = .007, respectively). In contrast, Positive Decisional Balance score, Knowledge score, and Barrier score were not associated with stage of change. Post-hoc analysis of the individual items on the Decisional Balance questionnaire revealed that 55.6% of women in the Precontemplation group believed that a mammogram was not needed if a clinical breast exam was preformed compared to 18.5% of the women in the Action/Maintenance group and none in the Contemplation group. (Chi-square = 7.292, d.f, = 2, p = .026.) Furthermore, all of the women in the Precontemplation group indicated that they would not have a mammogram if the doctor seemed to doubt that one was needed. In contrast, only 57.1% of the Contemplation group and 40.7% of the Action/Maintenance group endorsed this negative attitude (Chi-square = 7.892, d.f. = 2, p = .019.) No other significant differences were found among the groups. Lending further support for this observation, post-hoc analysis of the 13-item Barriers questionnaire also disclosed a significantly greater propensity for women in the Precontemplation group to not have a mammogram if a clinical breast exam was performed or if the doctor doubted the need for one.

## Discussion

We found that the proportion of women who received mammograms in the past year was lower than the rate reported for the general population in California (41.3% versus 60.5% for ages 40–64) [62] and more closely aligned with screening rates for minority populations living in the same community [63-65]. The low rate of screening may be considered even more disconcerting in view of the fact that nearly all of the women reported that they had insurance (88.8%), a primary care physician (91.1%), and annual physical examinations (73.3%). This low screening rate places them at a considerably greater risk of having a breast cancer discovered at a late stage, thereby supporting the first hypothesis.

As further support of the first hypothesis, only 41% endorsed having annual mammograms and only 63% of women reported undergoing mammography within the past two years (as defined by being in the Action or Maintenance stage), a rate lower than that seen in the general population and consistent with other research examining preventive care in women with severe mental illness [11,66]. One study found that 62% of their sample of women with schizophrenia had mammograms in the last two years [11]. Another study reported that women with schizophrenia and a low-income comparison group received similar preventive services, but the rates of mammography were not directly reported. It is not clear from this study, whether these women with schizophrenia received breast cancer screening at less than the recommended rates because of low income, lack of a physician referral, or due to their psychiatric illness. Further research is warranted to develop effective interventions.

Data from this study also supported the second hypothesis. We found some support for the validity of the Health Belief and Stages of Change models for predicting mammography adherence in women with schizophrenia. From Champion and colleagues' [36] study of women without schizophrenia, those in the Action and Maintenance stage perceived significantly higher susceptibility, more seriousness, fewer barriers, and more benefits than those in the Precomptemplation or Comtemplation stages. We did not find significant differences on measures of knowledge and benefits of breast cancer screening in our sample. However, women in the Precomtemplation stage did report more barriers, which differed from the other two stages of change categories, but at the trend level only. We did, however, find support for the shift of Decisional Balance over the stages of change, as predicted by the Stages of Change model. Interestingly, positive attitudes did not vary across stage of change. Negative attitudes, however, were lower in the women who were currently adhering to recommended mammography screening guidelines, suggesting that interventions with this group might be more effective if focused on ways of reducing negative attitudes.

The third hypothesis was also supported. A pattern was observed between knowledge and benefits of, attitudes toward, and barriers to breast cancer screening by stage of change in women with schizophrenia that has been reported in non-psychiatric groups. The more negative attitudes toward mammography were found in women with schizophrenia who were categorized as being in the Precontemplation stage, a finding that is consistent with the Stage of Change Model.

### Limitations

The findings from this study must be generalized with caution because the sample is small, the study participants were stable outpatients on antipsychotic medication, and only one large metropolitan region was studied. Moreover, a self-selection bias cannot be overlooked; by agreeing to participate in research, these women have set themselves apart from those women with schizophrenia who refused the invitation to participate. Finally, this was a cross-sectional study of stages of changes for mammography. According to the theory, a longitudinal study is needed to verify the shift in decisional balance over time.

## Conclusion

This study found that rates of breast cancer screening in women with schizophrenia were below the recommended guidelines, which may put them at greater risk for a late-stage breast cancer diagnosis. Moreover, this exploratory study of the application of the HB and SOC models to mammography in older women with schizophrenia suggests that these models may be useful for understanding and promoting the use of breast cancer screening in this population. Unlike the findings for reducing substance use behaviors in persons with schizophrenia, these results suggest the HB and SOC models may have utility in promoting some positive health behaviors in women with schizophrenia.

## Competing interests

The author(s) declare that they have no competing interests.

## Authors' contributions

LAL conceptualized and designed the study, analyzed and interpreted the data, and wrote the manuscript. EGW substantially contributed to data collection, and assisted with data analysis and interpretation, as well as wrote drafts of the manuscript. GRS significantly contributed to the writing and revising intellectual content of the manuscript. All authors read and approved the final manuscript.

## Pre-publication history

The pre-publication history for this paper can be accessed here:


